# Research on Optimized Design of In Situ Dynamic Variable-Aperture Device for Variable-Spot Ion Beam Figuring

**DOI:** 10.3390/mi16080849

**Published:** 2025-07-24

**Authors:** Hongyu Zou, Hao Hu, Xiaoqiang Peng, Meng Liu, Pengxiang Wang, Chaoliang Guan

**Affiliations:** 1 College of Intelligent Science and Technology, National University of Defense Technology, Changsha 410073, China; zou_hy2001@163.com (H.Z.); pengxiaoqiang@nudt.edu.cn (X.P.); meng_liu150@163.com (M.L.); wangpengxiang@nudt.edu.cn (P.W.); chlguan@nudt.edu.cn (C.G.); 2 National Key Laboratory of Equipment State Sensing and Smart Support, Changsha 410073, China

**Keywords:** variable aperture, variable spot, ion beam figuring, diaphragm sheets, FOC, removal function

## Abstract

Ion beam figuring (IBF) is an ultra-high-precision surface finishing technology characterized by a distinct trade-off between the spot size of the removal function and its corresponding figuring capabilities. A larger spot size for the removal function leads to higher processing efficiency but lower figuring ability. Conversely, a smaller spot size results in higher figuring ability but lower efficiency. Adjusting the spot size of the removal function using tools with an aperture is a possible approach. However, existing variable-aperture tools have certain limitations in IBF processing. To leverage the advantages of both large and small spot sizes for the removal function during IBF processing, an in situ dynamic beam variable-aperture device has been designed. This device optimizes the parameters of diaphragm sheets and employs FOC for dynamic aperture adjustment. Simulations show that 12 numbers of 0.1 mm-thick sheets minimize removal function distortion, with the thermal strain-induced area variation being <5%. FOC enables rapid (≤0.45 s full range) and precise aperture control. Experiments confirm adjustable spot sizes (FWHM 0.7–17.2 mm) with Gaussian distribution (correlation >96.7%), operational parameter stability (relative change rate ≤5%), and high repeatable positioning precision (relative change rate ≤3.2% in repeated adjustments). The design enhances IBF efficiency, flexibility, and accuracy by enabling in situ spot size optimization, overcoming conventional limitations.

## 1. Introduction

Ultra-high-precision figuring technology [1,2,3] has seen significant advancements in recent decades, becoming a critical technology in modern manufacturing. These developments have not only propelled progress in nanofabrication [4] but are also extensively applied in high-tech industries such as optical elements [3,5], semiconductor devices [6], and aerospace [7]. Among the various ultra-high-precision figuring techniques, IBF is widely regarded as the “final step” due to its unique advantages, including precise atomic-level material removal, an excellent surface finish, and non-contact figuring [8,9,10,11]. During the process, the discharge chamber of the ion source emits electrons to ionize the incoming argon gas, thereby generating plasma. The ions then diffuse into the screen grid, forming an ion sheath that passes through the screen and acceleration grids, where they are accelerated. The resulting ion beam is confined by a diaphragm with an aperture, which bombards the surface of the optical element, transferring energy to the surface lattice atoms, detaching them, and facilitating material removal [12].

IBF processing follows the constant current optical surface (CCOS) principle, which addresses the dwell time by distributing surface shape errors and selecting the appropriate removal function. This method allows for the calculation of the machine tool’s velocity at specific coordinate points [13,14]. The spot sizes of the removal function are inherently correlated with the figuring capability; a smaller spot size enhances the figuring ability and cutoff frequency but reduces efficiency, while a larger spot size improves figuring efficiency but compromises the figuring capability relatively [15,16,17]. Consequently, in IBF processing, surface error of optical element convergence is typically achieved using an iterative method. When surface error is high, a larger spot size for the removal function is used, and as the error diminishes, the spot size for the removal function is reduced to achieve better accuracy [18,19,20,21].

Current methods for adjusting removal function spot sizes include grids, ion diaphragms, and electromagnetic lenses. Grids modify spot sizes by altering the mesh diameter, grid pitch, or grid plate curvature [22,23,24]. While Liao achieved a 6 mm FWHM removal function via curvature and mesh adjustments [25], and Lu simulated the effects of the thickness, curvature, pitch, and aperture [26,27], grids are fundamentally limited to a minimum FWHM of 4 mm. Larger spots fail to meet ultra-high-precision optics requirements. Crucially, changing grid parameters necessitates opening the vacuum chamber for replacement, a process that takes approximately three hours, which prevents rapid spot size changes and impairs efficiency. Ion diaphragms physically block part of the ion beam flux, and aperture adjustment requires replacing the diaphragm [17,28,29]. Optimized grids and diaphragms enable IOM technology to achieve a 0.5 mm FWHM removal function [30,31]. NUDT uses a clamping diaphragm for smaller spots, maintaining less than 5% removal function parameter change during operation [17,25]. Although diaphragms allow smaller spots than grids and have simpler structures, spot size adjustment still requires vacuum chamber cycling and aperture replacement. Critically, each time the ion source is shut down and then started up again, the change rate in the volume removal efficiency of the removal function exceeds 15% [32]. A magnetic pedestal device enables five-axis machine tools to mount or dismount diaphragms in situ for aperture changes. Leybold switched up to five values [33], and Guo achieved 1–8 mm of adjustment. However, this method requires machine tool rotation unavailable on three-axis systems, necessitates repositioning after each change, offers limited aperture variety, and lacks flexibility. Electromagnetic lenses enable in situ dynamic adjustment by varying the lens coil current to control ion beam focusing and dispersion via the magnetic field strength [34,35,36]. Qiao used a single lens to reduce the FWHM from 12.1 mm to 10.1 mm [37]. Zhou employed dual diaphragms and three lenses to reduce divergence and improve control [38]. Zhang designed an energy filter using voltage to reject ions, adjusting the beam sizes [39]. While the adjustment time was shorter compared with the alteration of ion diaphragms, the reduction in FWHM was modest. Additionally, the process is complex, and operation in strong magnetic fields poses a risk of interference with sensitive electronics, potentially compromising stability.

Despite these studies aimed at optimizing the spot size of removal function adjust methods, existing technologies still fall short in effectively achieving in situ dynamic adjustment of the aperture values. Table 1 outlines our device’s performance goals, which are benchmarked against current limitations and scientific requirements in ultra-high-precision figuring. Traditional grids are constrained by their structure to a minimum FWHM of 4 mm, whereas ion diaphragms can achieve smaller beam diameters but necessitate cycling the vacuum chamber. Our design aims to achieve an FWHM range of 1–15 mm, corresponding to an aperture adjustment range of 1–20 mm, enabling the removal function size to meet the figuring requirements of corresponding error frequency bands [40,41] and thus balancing figuring capability and efficiency. Regarding the adjustment time, previous studies have not addressed practical requirements. For future applications involving error frequency band distribution and completing variable removal function figuring within a single machining cycle, combined with the dynamic response speed of ion beam machines [32], the adjustment time for the entire range should be less than 0.5 s. When the removal function exhibits a rotationally symmetric Gaussian distribution, the figuring capability is equivalent in all directions [42], making it the most ideal removal function. To avoid the influence of the aperture design on the shape of the removal function, the deviation from the rotationally symmetric Gaussian distribution must be limited to within 95%. Furthermore, the stability of figuring tools is fundamental to the ultra-high-precision figuring of optical elements [25,43]. Previous research has shown that in machining methods based on the CCOS principle, when the relative change rate of the removal function is within 5%, the impact on the machining process is minimal. Therefore, the relative change rate of the removal function should be within 5%. The positioning repeatability of the device affects the recurrence accuracy of the removal function. To avoid the figuring process, the recurrence accuracy of the removal function parameters must be within 5%. To meet these goals, this paper develops an in situ adjustment device based on ion diaphragms which enables direct adjustment of the aperture value without vacuum environment disruption. By optimizing key structures and employing the FOC method to enhance dynamic response and aperture adjustment accuracy, compliance with IBF requirements is ensured.

## 2. Structural Design and Control

### 2.1. Device and Working Principle

**Figure 1:**
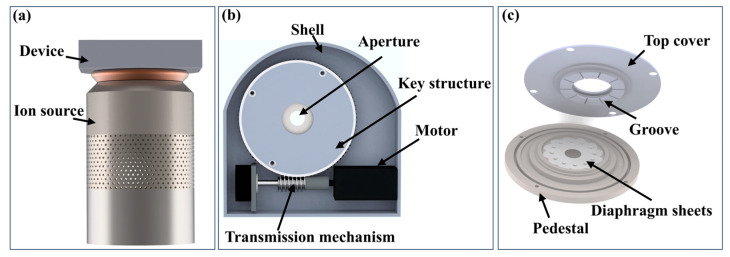
Schematic structure of the in situ dynamic variable-aperture device: (**a**) installation position; (**b**) overall structure; and (**c**) key structure.

The in situ dynamic variable-aperture device is installed at the upper end of the ion source, as shown in Figure 1a. It consists primarily of a pedestal, multiple double-arc diaphragm sheets, a top cover, transmission mechanisms, a motor, and a shell. The aperture is located between the pedestal and the top cover, consisting of several overlapping diaphragm sheets, as shown in Figure 1b,c.

The working principle is as follows. When the motor is activated, the top cover rotates via the transmission mechanisms. The groove in the top cover then guides the rotation of the diaphragm sheets on the pedestal. This rotational movement enables the aperture value to adjust within the range of 1–20 mm. By controlling the interception, the spot size of the removal function can be dynamically adjusted.

### 2.2. Key Structural Design

**Figure 2:**
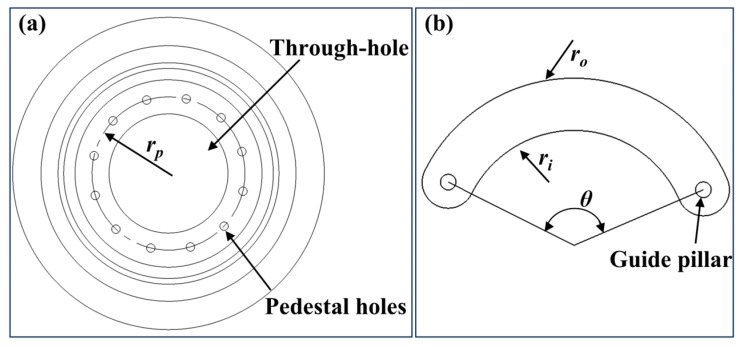
Dimensions of key structure: (**a**) pedestal and (**b**) double-arc diaphragm sheet.

The configuration of the parameters and mounting structure of the double-arc diaphragm sheet constitutes the fundamental design parameters of the device. Each double-arc diaphragm sheet is equipped with a guide pillar at both ends. One guide pillar is mounted in the pedestal hole, while the other is placed in the guide groove of the top cover. To ensure that the rotations of the multiple diaphragm sheets do not interfere with each other, the radius of the circle formed by the centers of the multiple holes on the pedestal (Figure 2a) and the central angle of the two guide pillars (Figure 2b) are designed as follows:

(1) $$\left\{\begin{array}{l} r_{p}=\frac{R_{\max}+\sqrt{7R_{\max}^{2}-3R_{\min}(2R_{\max}-R_{\min})}}{3}\\ \theta =2\arcsin\frac{r_{p}+R_{\max}}{2r_{p}} \end{array}\right.$$

where *r_p_* is the radius of the circumference of the center of the multiple holes on the pedestal, *θ* is the central tension angle between the two guide pillars, *R*_max_ is the maximum radius of the aperture, and *R*_min_ is the minimum radius of the aperture.

The diaphragm sheets are curved strips with arcs. Depending on the required aperture value range, when the aperture reaches its maximum value, the diaphragm sheets are rotated outward to their maximum limit, with the inner arc fitting to the circle of the maximum aperture value. It is also essential to avoid additional beam leakage, which could occur if the diaphragm cannot completely cover the edge of the through hole in the center of the base when the aperture is set to its minimum value. Therefore, the design equations for the inner and outer arc radius are as follows:

(2) $$\left\{\begin{array}{l} r_{i}=R_{\max}\\ r_{o}=2r_{p}-R_{\max} \end{array}\right.$$

where *r_i_* is the inner arc radius and *r_o_* is the outer arc radius.

In order to account for the stacked fit of the diaphragm sheets during mounting and the shape of the aperture, the equation for determining the number of diaphragm sheets is as follows:

(3) $$n=\frac{360}{\arccos\frac{R_{\max}^{2}+(R_{\max}-R_{\min})-{r_{o}}^{2}}{2R_{\max}(R_{\max}-R_{\min})}-\arccos\frac{R_{\max}-R_{\min}}{2R_{\max}}}$$

where *n* is the number of diaphragm sheets.

In the IBF processing, the value of the aperture was set from 1 mm to 20 mm, and the number of double-arc diaphragm sheets was calculated to be 12 sheets with Equation (3).

### 2.3. Design of Control Method

To calculate the maximum radian of motor rotation in the range of aperture value transformation, the equation is

(4) $$\varepsilon =\frac{\pi \left|d_{2}-d_{1}\right|\lambda }{180k}$$

where *ε* is the radian, *d*_2_ is the target aperture value, *d*_1_ is the current aperture value, *λ* is the deceleration ratio, and *k* is the setting factor.

The reduction ratio of the transmission mechanisms is 4, with a coefficient of 0.2. The rotation angle is 13.6 radians when the aperture value of the device is adjusted from the minimum to the maximum, as calculated with Equation (4). Therefore, the device does not require a high motor speed during operation; it only needs to be controlled at a smooth and precise speed and position. To achieve this, the FOC method was chosen, with a triple closed-loop control system incorporating current, speed, and position feedback loops to ensure precise dynamic response and steady-state control.

**Figure 3:**
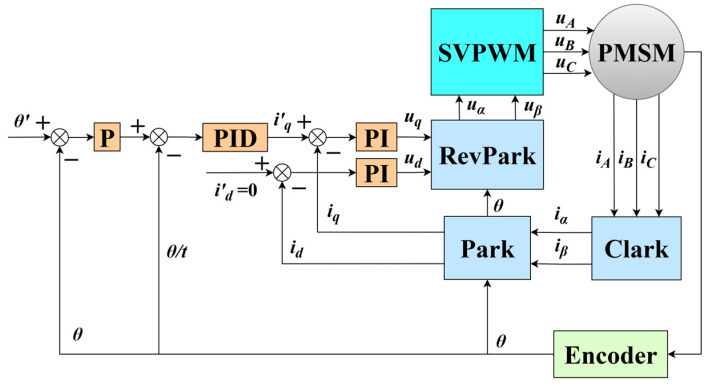
Block diagram of FOC control system.

As shown in Figure 3, the fundamental principle of the control system is outlined. The inner loop is the current loop, where the driver transmits the output current signal to each phase of the motor. This signal is then fed back for PI regulation, ensuring the output current closely matches the set current. The intermediate loop acts as the speed loop, regulated by a PID control unit, with the introduction of a differential term to enhance dynamic performance. The output speed of the motor is adjusted by fixing the speed signal to maintain speed accuracy during acceleration and deceleration. The outer loop is a position loop using only P regulation. The position error signal is derived by comparing the real-time position feedback from the encoder with the target position. This signal is processed by the P controller to generate a control output, which adjusts the speed loop and current loop, gradually bringing the motor to the target position.

## 3. Simulation Analysis

### 3.1. Simulation of Removal Function

**Figure 4:**
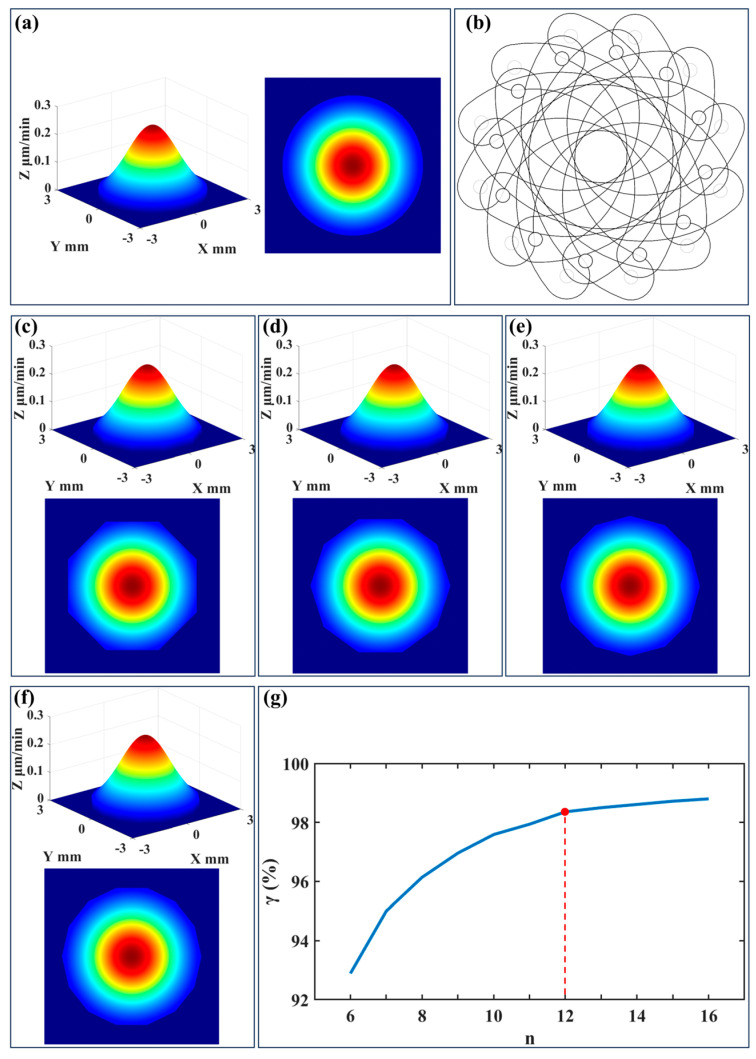
Simulation of removal function: (**a**) circular aperture; (**b**) schematic of the polygonal arc aperture; (**c**) *n* = 8; (**d**) *n* = 10; (**e**) *n* =12; (**f**) *n* = 14; and (**g**) simulation results.

In ultra-precision figuring, the Gaussian-type removal function is regarded as the most ideal one. In traditional processing, when an ion beam passes through a circular aperture, the resulting removal function typically exhibits a rotationally symmetric Gaussian distribution, as illustrated in Figure 4a. This rotationally symmetric Gaussian profile ensures ultra-high precision and uniform material removal during processing. However, the aperture in the in situ dynamic variable-aperture device does not adopt a standard circular shape. Instead, it consists of multiple overlapping double-arc diaphragm sheets, forming a polygonal arc aperture structure, as illustrated in Figure 4b. To investigate the effect of this non-standard aperture structure on the shape of the ion beam removal function, this study performed simulation analyses on the removal function profiles after the ion beam passed through apertures with a diameter of 5 mm formed by different numbers (8–14) of diaphragm sheets. The simulation results, depicted in Figure 4c–f, show that as the number of diaphragm sheets increased, the shape of the removal function gradually approached a rotationally symmetric Gaussian distribution.

To further quantify the impact of the number of diaphragm sheets on the shape of the removal function, the correlation coefficient method is employed. It examines the correlation between the removal functions generated by polygonal arc apertures with different sheet numbers and the standard circular aperture:

(5) $$\gamma =\frac{\sum_{x}\sum_{y}(I_{c}(x,y)-I_{c2})(I_{p}(x,y)-I_{p2})}{\sqrt{\left(\sum_{x}\sum_{y}{\left(I_{c}(x,y)-I_{c2}\right)}^{2}\right)\left(\sum_{x}\sum_{y}{\left(I_{p}(x,y)-I_{p2}\right)}^{2}\right)}}$$

where *γ* is the correlation coefficient, *I_c_*(*x*,*y*) is the distribution of the removal function by the circular aperture, *I_p_*(*x*,*y*) is the distribution of the removal function by the polygonal arc aperture, and *I_c_*_2_ and *I_p_*_2_ are the average values.

The calculation results are shown in Figure 4g, indicating that the correlation with the standard circular aperture reached more than 98% when the number of diaphragm sheets reached 12, and then the correlation coefficient increased slowly. Due to design and mounting constraints, the number of diaphragm sheets could not be increased indefinitely. Therefore, when the number of diaphragm sheets reached 12, the shape of the removal function approximated a rotationally symmetric Gaussian distribution, meeting the figuring requirements, which aligned with the number of diaphragm sheets calculated by Equation (3).

### 3.2. Analysis of Simulation Using FEM

**Figure 5:**
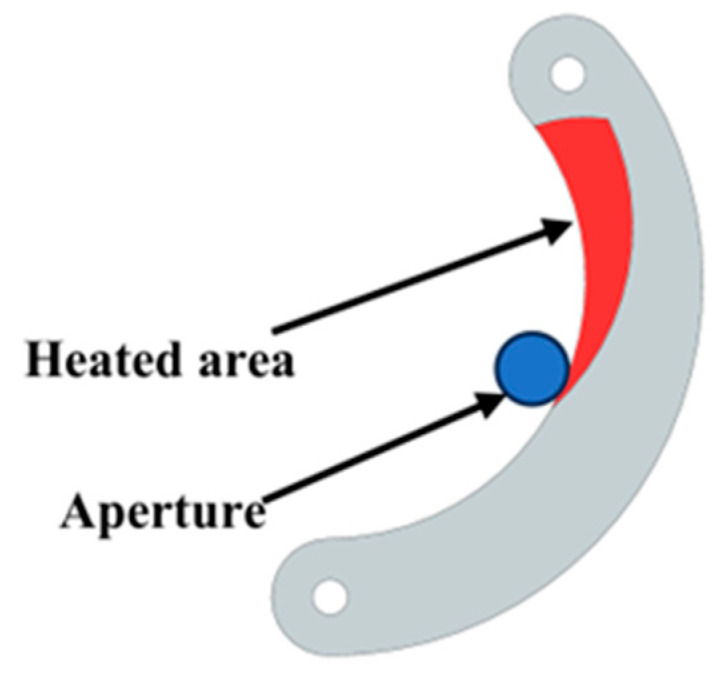
Schematic diagram of the heat area of the diaphragm sheet.

During operation of the device, the diaphragm sheet is subjected to the influence of the ion beam, which induces thermal strain and consequently leads to changes in the aperture area. The aperture value directly determines the flux of the ion beam, thereby affecting the parameters of the removal function. As shown in Figure 5, the red area represents the region of the diaphragm sheet exposed to the ion beam during the figuring process. When the aperture value is set to its minimum, the heated area of the diaphragm sheet reaches its maximum, resulting in the most significant thermal strain. Yuan [44] demonstrated through infrared thermography that the highest temperature on the surface of an object exposed to an ion beam can reach up to 100 °C. To evaluate the thermal strain in diaphragm sheets under ion beam exposure, finite element method (FEM) simulations were conducted using ANSYS 2022 R1. The simulation details were as follows.

The model represented a double-arc diaphragm sheet made of molybdenum (Mo), selected for its high thermal conductivity and low thermal expansion, which are critical for resisting ion beam-induced thermal distortion. The geometric dimensions aligned with the structural design in Equations (1) and (2), with the tested thicknesses ranging from 0.06 mm to 0.12 mm. The key thermophysical properties of the molybdenum were shown in Table 2.

One end of the diaphragm sheet was fixed to the pedestal, constraining all degrees of freedom, while the opposite end was left free to allow thermal expansion during aperture adjustment. A uniform heat flux was applied to the heated area of the diaphragm sheet, resulting in a maximum surface temperature of 100 °C. To balance accuracy and computational efficiency, a tetrahedral mesh with adaptive refinement was used. The minimum element size was 0.05 mm in the heated region and 0.2 mm in the unheated regions. The simulation process integrated steady-state thermal analysis and static structural analysis.

**Figure 6:**
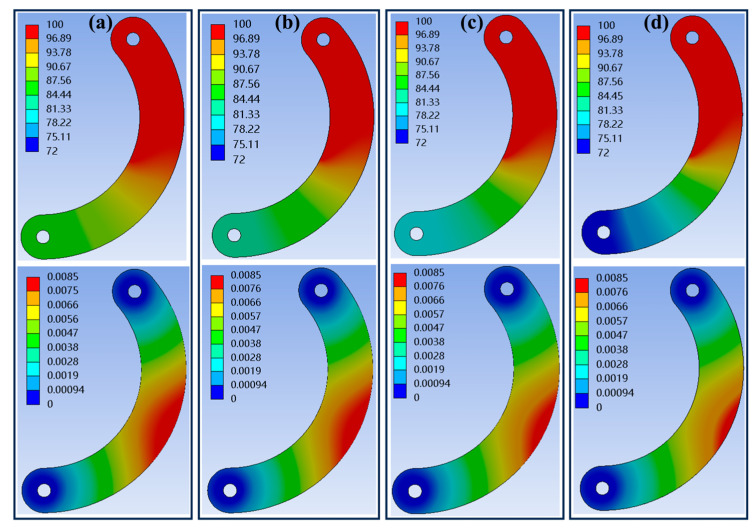
Heat and thermal strain of diaphragm sheet: (**a**) thickness of 0.06 mm; (**b**) thickness of 0.08 mm; (**c**) thickness of 0.10 mm; and (**d**) thickness of 0.12 mm.

The simulation results for the heat distribution and thermal strain of the diaphragm sheet are presented in Figure 6. As the thickness increased, the temperature at the unheated end of the diaphragm sheet gradually decreased, and the thermal strain at the diaphragm sheet became progressively smaller. The thermal strain measurements at the aperture location using the software probe function are shown in Table 3. The aperture area of the diaphragm, after accounting for thermal strain, is as follows:

(6) $$S=\pi {\left(\frac{2\pi R_{\min}+n\Delta l}{2\pi }\right)}^{2}$$

where *S* is the area of the diaphragm sheet after thermal strain, *R*_min_ is the minimum radius of the aperture, *n* is the number of diaphragm sheets, and Δ*l* is the thermal strain value.

The amount of variation in the area of the aperture is

(7) $$\delta =\frac{S-\pi R_{\min}^{2}}{\pi R_{\min}^{2}}$$

This could be calculated that when the thickness was 0.1 mm. The variation of the area of the aperture did not exceed 5%, which is considered to meet the requirements.

### 3.3. Control System Simulation and Analysis

The simulation model was constructed using MATLAB R2022b/Simulink software. The process began with the development of the motor model, followed by the creation of the signal transformation module, which was designed according to the control block diagram shown in Figure 3. Subsequently, the controllers for the current loop, speed loop, and position loop were developed and connected sequentially.

**Figure 7:**
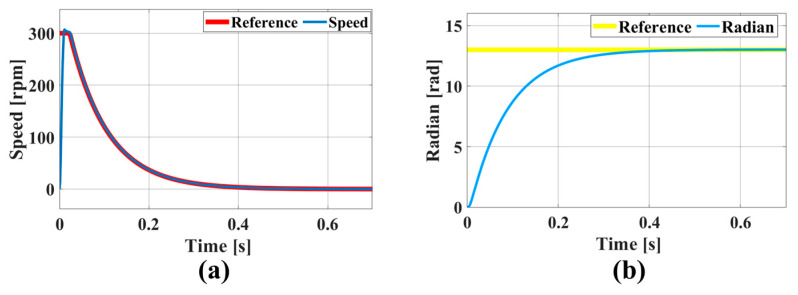
Simulation results for FOC control system: (**a**) speed and (**b**) position.

It is important to note that the maximum speed of the motor was limited to 300 rpm. The dynamic adjustment of the device’s aperture value, from its maximum to the minimum, is demonstrated in Figure 7a. The results show that the system could quickly adjust the actual speed to match the reference rotational speed. As the reference speed decreased, the actual speed decreased smoothly, with no significant oscillation or overshooting during the adjustment process. The position simulation results, shown in Figure 7b, reveal that the reference position was reached swiftly and accurately within 0.45 s. The entire position change process was characterized by smoothness, with a maximum position error of 0.01 rad.

In the context of IBF processing, the use of a variable-spot removal function approach, which is influenced by either regional error or frequency band error, imposes stringent dynamic performance criteria on the system. The requirement is a rapid and consistent response within brief intervals. The variable-aperture values were limited to a maximum of 5 mm.

**Figure 8:**
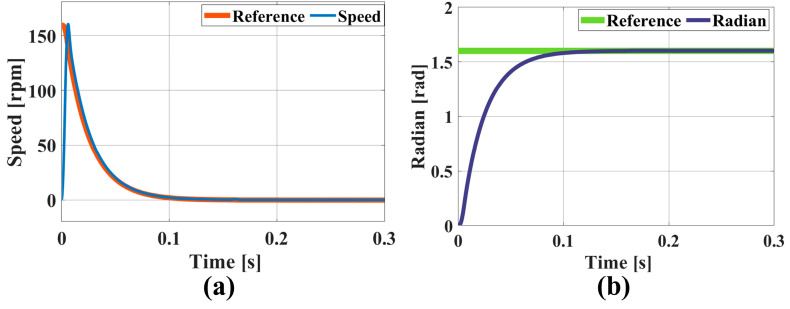
Simulation results for FOC control system over brief intervals: (**a**) rotational speed and (**b**) position.

Upon entering the reference position into the simulation model, the rotational speed change curve is shown in Figure 8a. The simulation results demonstrate that the rotational speed quickly tracked the reference speed within 0.11 s, and the entire adjustment process was smooth. The position simulation results are depicted in Figure 8b, where the position has a maximum position error of 0.008 rad, stabilizing at the reference position within 0.11 s.

## 4. Experiment Results

**Figure 9:**
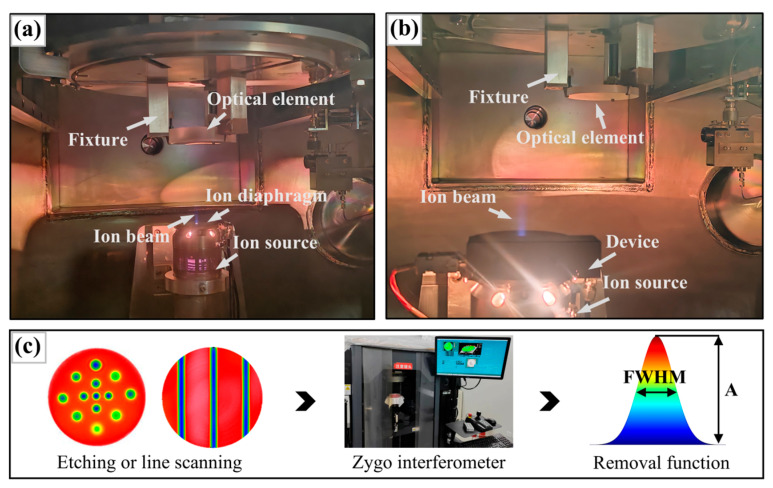
Experimental set-up and data collection method: (**a**) set-up with ion diaphragm; (**b**) set-up with device; and (**c**) data collection procedure.

The experimental set-up included an ion diaphragm, an ion source, and a fused silica optical element fixed in a fixture (Figure 9a). As shown in Figure 9b, the in situ dynamic variable-aperture device was mounted on top of the ion source. During these experiments, the ion source was moved to etch or scan the surface of the optical element. The surface shape data of the optical element were subsequently measured using a wavefront interferometer following the point etching or line scanning experiments. The parameter information associated with the removal function was then calculated by subtracting the surface shape data obtained after etching or scanning from the initial surface shape data of the optical element and extracting the relevant etching points or grooves.

### 4.1. Verification of Spot Size and Shape in Removal Function

**Figure 10:**
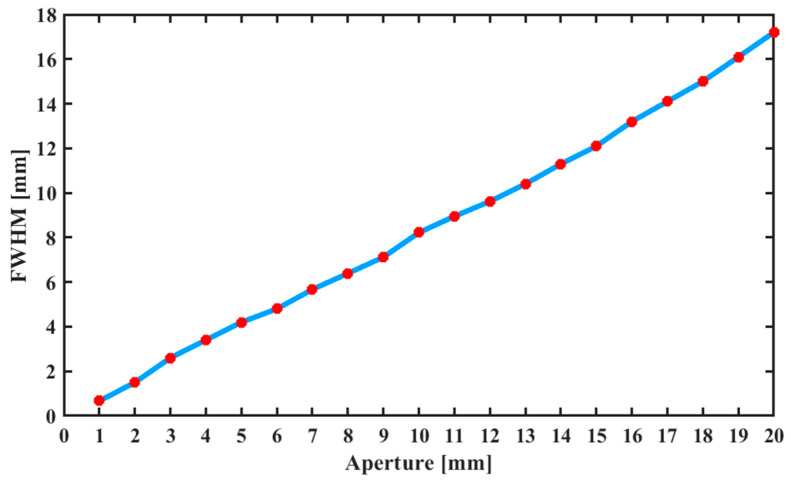
Relationship between the aperture value and FWHM of the removal function.

To validate the FWHM adjustment range of the removal function when the aperture value was within 1–20 mm, point etching experiments were conducted by adjusting the aperture in 1 mm increments. The surface of the optical element was etched at different positions for 1 min. A total of 20 test points were evaluated, and the FWHM values of the corresponding removal functions were recorded. As shown in Figure 10, within the aperture diameter range of 1–20 mm, the FWHM of the removal function varied from 0.7 mm to 17.2 mm, which meets the design specifications of the equipment. This range addresses the limitations of traditional tools in IBF; traditional grids and ion diaphragms require vacuum chamber operations for adjustment. The device, via in situ dynamic adjustment, enables continuous tuning within this range and supporting balanced efficiency and precision in a single machining cycle.

**Figure 11:**
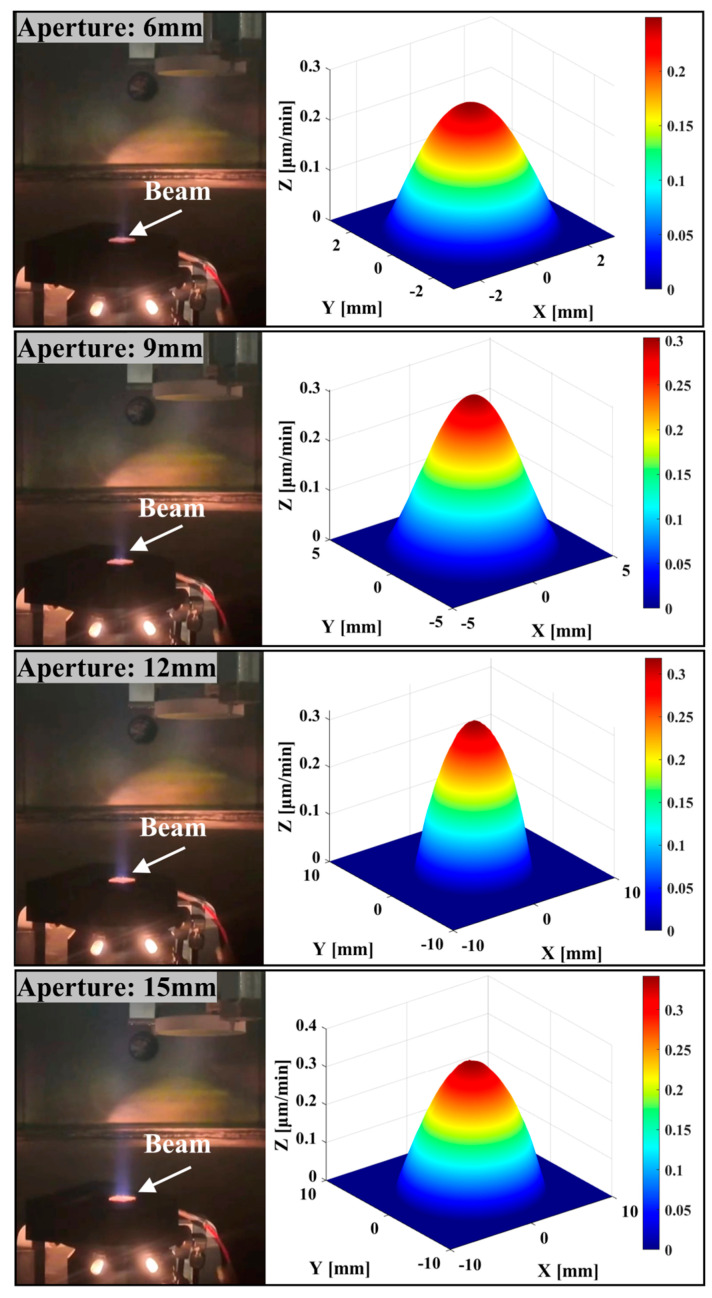
Beam control of device and removal function shape at different apertures.

Additionally, using the 12 diaphragm sheets with the polygonal arc aperture obtained from the above simulation experiments, the correlation between the shape of the removal function and the standard rotationally symmetric Gaussian type was investigated during actual ion beam operation. The control effects of the in situ dynamic variable-aperture device on the ion beam were recorded for aperture values of 6 mm, 9 mm, 12 mm, and 15 mm, and the corresponding removal functions were calculated (see Figure 11). The results demonstrate that the device effectively intercepted the ion beam after the ion source was activated, achieving the expected beam size adjustment effect. The correlation coefficient between the removal function and the rotationally symmetric Gaussian distribution (calculated using Equation (5)) exceeded 96.7%, satisfying the requirements for the removal function shape in IBF.

### 4.2. Stability of Removal Function

**Figure 12:**
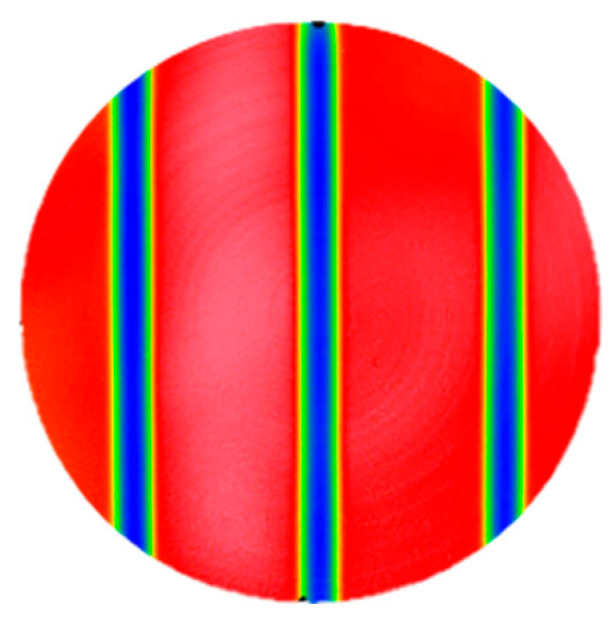
Schematic of line scanning experiment.

IBF processing requires high stability of the removal function. To investigate the impact of the device on the parameters of the removal function during IBF processing, line scanning experiments were conducted. In these experiments, the controlled ion beam continuously etched three grooves on the surface of the optical element at a speed of 4 mm/min, as shown in Figure 12. The total experiment duration was approximately 65 min. The peak removal efficiency and the volume removal efficiency of the etched groove cross-section were calculated using Equation (8) [42].

(8) $$\left\{\begin{array}{l} A=\frac{v}{\sqrt{2\pi }\sigma }A_{s}\\ B=v\sqrt{2\pi }\sigma A_{s} \end{array}\right.$$

where *A* is the peak removal efficiency, *B* is the volumetric removal efficiency, *v* is the velocity of movement degree, and *A_s_* and *σ* are the fitting parameters for the groove crosssection.

**Figure 13:**
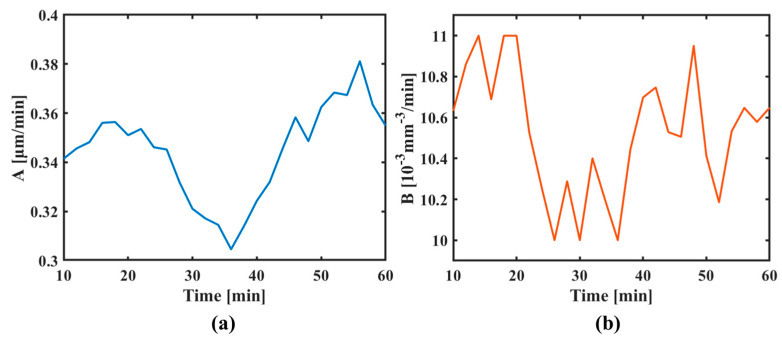
Results of line scanning experiments: (**a**) peak removal efficiency and (**b**) volume removal efficiency.

The position at each cross-section was converted to time, and the curves of the peak removal efficiency and volume removal efficiency as a function of time were plotted. The relative change rates in time between 10 and 60 min were analyzed, as shown in Figure 13. The relative change rate in the peak removal efficiency and volume removal efficiency throughout the experiment was found to be within 4.8%, demonstrating excellent consistency among the three grooves. Therefore, the results of the line scanning experiments confirmed that the removal function exhibited good stability even after using the device.

### 4.3. Comparative Experiment for Recurrence Accuracy of Removal Function

**Figure 14:**
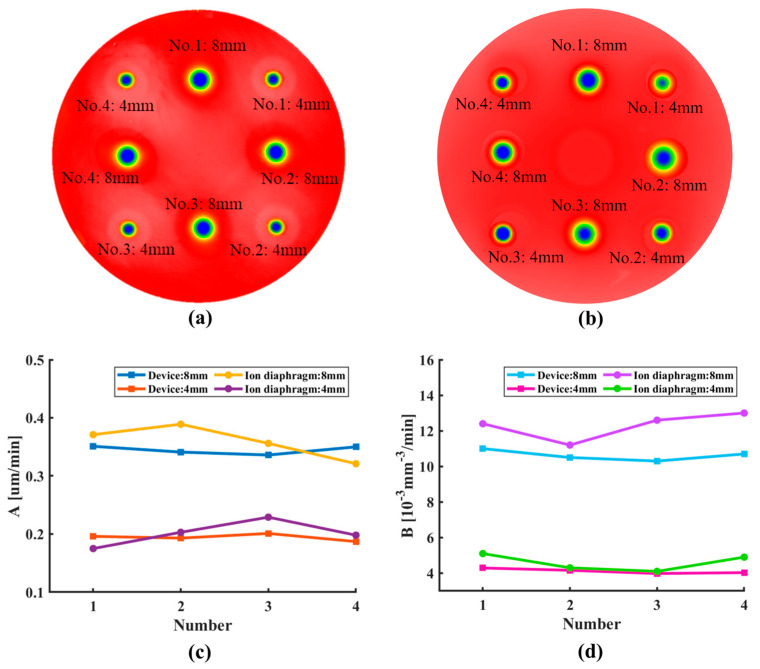
Recurrence accuracy of removal function’s comparative experiments: (**a**) schematic of the etching sequence using the device; (**b**) schematic of the etching sequence using the ion diaphragms; (**c**) peak removal efficiency; and (**d**) volume removal efficiency.

To verify the recurrence accuracy of the in situ dynamic variable-aperture device’s removal function after adjusting the aperture value, the following experiment was conducted. First, the aperture of the device was set to 8 mm, and the optical element surface was etched for 1 min. Subsequently, the aperture was adjusted to 4 mm, and etching was performed at different positions for 2 min. Then, the aperture was reset to 8 mm, and point etching was continued at new positions on the optical element surface. The entire process involved alternating between 8 mm and 4 mm apertures for a total of eight point-etching experiments, as shown in Figure 14a.

A comparative experiment was performed using the set-up without the device (as shown in Figure 9a) to investigate the relative change rate of the removal function during repeated ion source shutdown and startup. In this experiment, an 8 mm ion diaphragm was first used, with the ion source repeatedly shut down and started up (cooled for 1 h after each shut down and stabilized for 20 min after startup), accompanied by point etching at different positions on the optical element for a total of four times. This procedure was then repeated using a 4 mm ion diaphragm, as illustrated in Figure 14b.

Figure 14c,d presents the statistical analysis of the relative change rate in the peak removal efficiency and volume removal efficiency of the post-etching removal function. The results show that when using an ion diaphragm and requiring repeated ion source shutdown and startup (under the condition of opening and closing the vacuum chamber), the relative change rate of the peak removal efficiency remained within 13.9%, and that of the volume removal efficiency remained within 10.7%. In contrast, the device eliminated the need for ion source shutdown and startup. By maintaining continuous operation, it controlled the relative change rates of the peak and volume removal efficiencies within 3.2% and 2.9%, respectively. This recurrence accuracy directly ensured reliable iterative error correction in IBF, an advantage over traditional methods.

## 5. Conclusions

To leverage the advantages of both small and large spot sizes for the removal function in IBF processing, a device was developed that enables in situ dynamic adjustment of the aperture value. This device allows the spot size of the removal function to be dynamically varied during IBF processing to meet the requirements for ultra-high precision. The key conclusions of this study are as follows:

(1) The device enabled continuous aperture adjustment from 1–20 mm, directly corresponding to a removal function full-width at half-maximum (FWHM) range of 0.7–17.2 mm. The device enabled in situ dynamic tuning, overcoming traditional IBF tool limitations and achieving efficient, precise figuring in one cycle.
(2) Using the FOC system, the device completed full-range aperture adjustments (1–20 mm) within 0.45 s and small-range adjustments (≤5 mm) within 0.11 s. This eliminates the hours-long vacuum chamber cycling required by traditional methods, meeting the real-time dynamic response demands of IBF for frequency band error correction.
(3) With optimized double-arc diaphragm sheets, the removal function maintained a rotationally symmetric Gaussian distribution with a correlation coefficient exceeding 96.7%, surpassing the design target of 95%. This high conformity ensures uniform material removal in all directions, which is critical for ultra-high-precision optical fabrication.
(4) Thermal strain analysis using the finite element method (FEM) confirmed that the 0.1 mm-thick molybdenum diaphragm sheets exhibited aperture area variation below 5% under ion beam exposure. Line scanning experiments over 50 min further validated the relative changes in peak and volumetric removal efficiency to be within 4.8%, demonstrating operational stability.
(5) Repeated aperture adjustments yielded relative change rates within 3.2% for the removal function parameters, significantly outperforming traditional ion diaphragms (13.9% and 10.7% changes in peak and volumetric efficiency, respectively). The device’s in situ adjustment capability eliminates ion source shutdowns and vacuum chamber cycling, ensuring consistency in iterative processing and exceeding the 5% recurrence accuracy requirement.

## Figures and Tables

**Table 1:** Device performance goals.

Parameters	Performance Goals
Aperture value range [mm]	1–20
Removal function FWHM [mm]	1–15
Adjustment time [s]	0.5
Gaussian correlation	95%
Stability (relative change)	5%
Recurrence accuracy (relative change)	5%

**Table 2:** Material properties.

Material Property	Value
Thermal conductivity [W/(m·k)]	138
Coefficient of thermal expansion [K^−1^]	5.2 × 10^−6^
Density [kg/m^−3^]	10,200
Specific heat capacity [J/(kg·k)]	250

**Table 3:** Thermal strain results at the location of the diaphragm aperture.

Thickness (mm)	0.06	0.08	0.1	0.12
Thermal strain value (mm)	0.00665	0.00657	0.00644	0.00630

## Data Availability

The data presented in this study are available upon request from the corresponding author. The data are not publicly available because they are part of an ongoing study.
